# 351. Factors Predicting Readmission within 90 days in Patients Receiving Outpatient Parenteral Antibiotic Therapy

**DOI:** 10.1093/ofid/ofad500.422

**Published:** 2023-11-27

**Authors:** Kyle Smola, Julia Silliman, Minh Ho, Amanda Mercurio

**Affiliations:** University of Central Florida College of Medicine, Orlando, Florida; University of Central Florida College of Medicine, Orlando, Florida; Orlando VA Healthcare System, Orlando, Florida; Orlando Veterans Affairs Medical Center, Orlando, Florida

## Abstract

**Background:**

Outpatient Parenteral Antibiotic Therapy (OPAT) involves administering antibiotics outside hospitals via non-oral routes. Success depends on well-reasoned selection of patients for this treatment modality and proper antibiotic stewardship to avoid serious complications.

**Methods:**

We created a de-identified dataset (n=295) from a retrospective chart review of veterans receiving OPAT at the Orlando VA Medical Center (ORL-VAMC) in 2020. We included patients aged 18 or older who underwent OPAT therapy at ORL-VAMC with at least one antibiotic administration between January 1 and December 31, 2020. Inclusion also required compliance with follow-up and laboratory monitoring for at least 90 days after starting outpatient antibiotics. Of 364 patients receiving OPAT during 2020, 295 met the full criteria, with loss to follow-up and absent labs as common reasons for exclusion. We examined patient demographics, medical history, diagnosis, and elements of treatment for associations with unplanned hospital visits within 90-days of OPAT initiation using Binary Logistic Regression modeling.

**Results:**

The average age within the dataset was 67.37 (SD=±11.38) years. Common treatment indications included Osteomyelitis (80/295, 27.12%) and Bacteremia (64/295, 21.69%). 40.96% (119/295) of patients had an unplanned hospital visit within 90 days, primarily due to unresolved symptoms. Our regression model identified Pyelonephritis/UTI (OR 2.26; 95% CI 1.02-5.41), Intra-Abdominal Infection (OR 3.81, 95% CI 1.15-17.45), Cephalosporin Antibiotics (OR 2.57, 1.39-4.91), Liver Disease (OR 2.26, 95% CI 1.02-5.41), and psychiatric illness (OR 1.74, 95% CI 1.06-2.87) as being significantly associated with unplanned hospital visits within 90 days.

**Conclusion:**

A majority (177/295, 60%) of patients did not need further care in the hospital setting after starting OPAT. Our study supports OPAT as an effective option for delivering IV antibiotic therapy when indicated. This study's external validity is limited by the overrepresentation of males in the sample. Nevertheless, these findings can help physicians better anticipate successful outcomes and select suitable patients for OPAT. Further research aimed at understanding the associations identified may offer improved clinical utility.

**Disclosures:**

**All Authors**: No reported disclosures

